# Stable prevalence of chronic back disorders across gender, age, residence, and physical activity in Canadian adults from 2007 to 2014

**DOI:** 10.1186/s12889-019-7395-8

**Published:** 2019-08-15

**Authors:** Adriana Angarita-Fonseca, Catherine Trask, Tayyab Shah, Brenna Bath

**Affiliations:** 1 Community Health and Epidemiology Department, University of Saskatchewan, Rm 3247 - E wing - Health Sciences Building, 104 Clinic Place, Saskatoon, Saskatchewan S7N-2Z4 Canada; 2 Facultad de Ciencias de la Salud, Grupo de Investigación Fisioterapia Integral, Universidad de Santander, Bucaramanga, Colombia; 3 Canadian Centre for Health and Safety in Agriculture (CCHSA), University of Saskatchewan, Rm 1226 - E wing - Health Sciences Building, 104 Clinic Place, PO Box 23, Saskatoon, Saskatchewan S7N-2Z4 Canada; 4 School of Rehabilitation Science, University of Saskatchewan, Suite 3400 - E wing - Health Sciences Building, 104 Clinic Place, Saskatoon, Saskatchewan S7N-2Z4 Canada; 5 School of Rehabilitation Science and Canadian Centre for Health and Safety in Agriculture (CCHSA), University of Saskatchewan, Rm 1340 - E wing - Health Sciences Building, 104 Clinic Place, PO Box 23, Saskatoon, Saskatchewan S7N-2Z4 Canada

**Keywords:** Epidemiology, Back pain, Spine, Regression analysis, Joinpoint, Trends

## Abstract

**Background:**

Chronic back disorders (CBD) are a global health problem and the leading cause of years lived with disability. The present study aims to examine overall and specific trends in CBD in the Canadian population aged 18 to 65 years.

**Methods:**

Data from the Canadian Community Health Survey (CCHS), a cross-sectional study, from 2007 to 2014 (8 cycles) were used to calculate CBD prevalence across gender, age, geographical area (urban/rural and ten provinces and northern territories), and physical activity levels. CBD was defined in the CCHS as having back problems, excluding fibromyalgia and arthritis, which have lasted or are expected to last six months or more and that have been diagnosed by a health professional. Prevalence of CBD using survey weights and associated 95% confidence intervals (95% CI) were calculated yearly using balanced repeated replications technique. Trend tests were calculated using joinpoint regressions; ArcGIS software was used for mapping.

**Results:**

Age-standardized CBD prevalence in 2007 and 2014 were 18.9% (95% CI = 18.4;19.5) and 17.8% (95% CI = 17.2,18.4), respectively. CBD prevalence was consistently higher in women, older age groups, rural dwellers, and people classified as inactive. Crude and age-standardized CBD prevalence decreased faster in people classified as physically active compared to those who were inactive (*p* < 0.006). Although CBD slightly decreased over time, no statistically significant trends were found overall or by gender, area of residence, province or level of physical activity. The prevalence of CBD remained consistently high in the province of Nova Scotia, and consistently low in the province of Quebec over the eight CCHS cycles.

**Conclusion:**

Despite prevention efforts, such as the Canadian back pain mass media campaign, CBD prevalence has remained stable between 2007 and 2014. Tailored prevention and management of CBD should consider gender, age, and geographical differences. Further longitudinal studies could elucidate the temporal relationship between potentially modifiable risk factors such as physical activity and CBD.

**Electronic supplementary material:**

The online version of this article (10.1186/s12889-019-7395-8) contains supplementary material, which is available to authorized users.

## Background

Back disorders encompass a variety of pathologies and symptoms (pain, discomfort, muscle tension, stiffness, etc.) in regions that may include thoracic, lumbar spine, pelvic girdle or a combination of regions [1]. Among back disorders, low back pain is the most common condition reported in primary care [2]. Low back pain can be defined as symptoms that occur from the twelfth rib to the gluteal fold [3]. Globally, lower back pain have been the leading cause of years lived with disability in 1990, 2007, and 2017 [4]. Further, the lifetime prevalence of low back pain varies from 50 to 85% [5], whilst the rate of 1-year first-ever episode of low back pain ranges between 6.3 and 15.4% [6].

Based on the duration of the symptoms, back disorders can be acute or chronic. When symptoms last less than six months such is classified as acute back disorders, otherwise it is chronic back disorders (CBD) [7]. Compared to acute back disorders, individuals with CBD in particular are more likely to use health services as well as experience reduced productivity and quality of life [8, 9]. In Canada, there has been some prevention and management efforts to address CBD and related disability such as the guide on the prevention of musculoskeletal injuries [10], public health campaigns [11], booklets designed for working population [12, 13] and mobile applications [14]. Notwithstanding, information about Canadian secular trends in CBD that may allow evaluation of the impact of such efforts at a population level is lacking.

Among eight published studies [15–22] evaluating CBD in the general population using the Canadian Community Health Survey (CCHS), the prevalence of CBD was lower when children (12 and over) were included [15], and higher in studies involving Canadians over 20 years [16–19]. CCHS data has also been used to investigate the effect of gender [17, 20–22], place of residence [16] and physical activity [16, 18, 20] in different cycles. However, the trends in CBD prevalence over time have not been investigated by variables such as age, gender, and geographical region. Furthermore, there is no known evidence regarding the distribution of CBD in Canada and its relation to potentially modifiable risk factors such as physical activity. Physical activity has been considered an important strategy both for individual and population-level prevention of CBD [23] as well as to manage or prevent recurrences of back pain [24]. The relationship between physical activity and CBD has been inconclusive. For example, high levels of physical activity in women can increase the risk of having low back pain [25]; conversely, adults that are inactive have a higher risk of having CBD compared to people who report moderate physical activity levels [26].

Examination of CBD trends would elucidate the magnitude of this problem in Canada. The exploration of population subgroups would additionally allow identification of groups more likely to have CBD, which may help the planning of targeted preventative approaches for specific population groups and ultimately decrease the proportion of people with CBD. Epidemiological studies conducted in Britain [27, 28], United States [29], Austria [30, 31], Sweden [32], and Finland [33] suggest that the prevalence of CBD has increased over time, while other studies in Finland [34, 35] and Germany [36] have shown only small changes over decades or even diminished prevalence [37]. Although most of the research to date has shown an increasing prevalence of CBD, the trends over time are unknown among adults in Canada. This information is relevant to monitoring the burden of CBD in Canada and may help to highlight where there is a greater need for prevention and rehabilitation services. Therefore, the purpose of this study was to describe overall time trends in CBD and trends in prevalence by gender, age, geographical location, and physical activity levels between 2007 and 2014 in Canadian working age population.

## Methods

### Study design, data source, and study population

The CCHS is a cross-sectional survey conducted by Statistics Canada biannually from 2000 to 2006, and annually from 2007 to date. The CCHS aims to provide regional health data across Canadian provinces. Each CCHS cycle targets people 12 years or older living in private dwellings. Members of the Canadian forces, institutionalized people, those living in remote regions of Canada as well as on reserves are excluded (< 3% of the national population) from CCHS sampling frames [38]. Participants are selected using a two-stage sample design. In the first stage, households are identified by one of three sampling frames: the area frame, the list frame of telephone numbers, and random digit dialing (RDD). The area frame was designed for the Labour Force Survey, and selects clusters of households using a probability proportional to size sampling method. The list frame of telephone numbers is an external list complementing the area frame, and RDD is used to randomly generate a set of numbers until the required sample size was reached. After a household is selected, the last stage is to randomly select a respondent from a list of possible respondents living in the household. Interviews are conducted either in person or by the telephone, by trained Statistics Canada representatives [38]. The present study involves secondary analysis of the CCHS data from 2007 to 2014, including individuals aged between 18 and 65 years, using the restricted microdata at the Saskatchewan Research Data Center. Those who did not respond to the CBD question were excluded from the analysis. The response rate and the final analyzed sample sizes for each cycle can be found in Table 1.

**Table 1:** Sample size and response rate by the Canadian Community Health Survey cycle

Year	Response Rate^a^			Unweighted sample size^b^				
	Household-level response	Person-level response	Combined response rate	Total	Chronic Back Disorders			Analyzed
					Yes	No	Missing	
2007	84.6	91.7	77.6	46,634	9724	36,845	65	46,569
2008	84.6	91.7	77.6	45,793	11,515	34,218	60	45,733
2009	81.3	90.0	73.2	42,483	8896	33,547	40	42,443
2010	80.7	88.6	71.5	42,928	8784	34,102	42	42,886
2011	79.5	87.8	69.8	43,156	8735	34,358	63	43,093
2012	77.3	86.7	67.0	41,415	8327	33,026	62	41,353
2013	76.6	87.2	66.8	42,052	8518	33,472	62	41,990
2014	75.1	87.4	65.6	40,663	8352	32,247	64	40,599

^**a**^Calculated for the complete surveys including people 12 years or older. ^b^People aged 18–65 years

### Variables

The main outcome of interest was whether or not respondents reported having back problems using the question: *Now I’d like to ask about certain chronic health conditions which you may have. We are interested in ‘long-term conditions’ that have lasted or are expected to last 6 months or more and that have been diagnosed by a health professional. Do you/Does (name) have back problems, excluding fibromyalgia and arthritis?* Respondents were classified as having a ‘chronic back disorder’ (CBD) if they responded ‘yes’ to this question.

Gender, age, geographical residence (urban/rural area, province or territory), and self-reported physical activity levels were used for stratification purposes. Respondents’ age was classified into three groups based on biologically and clinically relevant categories established in previous studies [16]: 18 to 34 years old, 35 to 49 years old, and 50 to 65 years old [39]. Area of residence was classified as urban or rural. Urban areas included communities with ≥10,000 people with a density of 400 or more people per square kilometer [40]. Province of residence was classified in 11 categories, representing the ten provinces plus the combined northern territories (Yukon, Northwest, and Nunavut Territories). Based on self-reported frequency of daily participation in transportation and leisure time physical activities, participants were pre-classified in the CCHS data based on calculated daily energy expenditure as active (> 3.0 kcal/kg/day), moderately active (1.5–3.0 kcal/kg/day), and inactive (< 1.5 kcal/kg/day) [41].

### Statistical analyses

#### Prevalence of CBD

The prevalence of CBD and the corresponding 95% confidence interval (95% CI) were calculated using Stata 14.0 [42]. Analyzing each survey cycle separately, yearly prevalence estimates were calculated by gender, age, geographical location (urban-rural area, and province or territory), and physical activity levels. In addition, overall crude and age-standardized prevalence by direct standardization against the 2016 Canadian Census [43] were calculated. The survey weights provided by Statistics Canada were applied to account for the unequal probability of selection [44, 45]. The balanced repeated replications (BRR) method using the pre-calculated bootstrap weights provided by Statistics Canada [46] was used to compute the standard errors and associated confidence intervals of the prevalence of CBD. BRR is a resampling technique used for robust variance estimation [47].

#### Outcome mapping

For the purposes of mapping and comparison across each years’ data, yearly CBD prevalence values were converted into three categories. The +/− 0.5 standard deviations (SD) [48] from mean was used to define the cut-off values (i.e., converting into z scores) [49, 50]: High prevalence: > 0.5 SD; moderate prevalence: between − 0.5 and 0.5 SD; low prevalence: < 0.5 SD. ArcGIS 10.5 software [51] was used to visualize the spatial distribution of CBD prevalence.

#### Joinpoint regression analysis

Trends in prevalence of CBD were assessed using joinpoint regression analysis (Joinpoint Regression Software, Version 4.6.0-April 2018; Surveillance Research Program of the US National Cancer Institute). This software selects the best-fitting and simplest piecewise continuous log-linear model through data across time. The Monte Carlo permutation test [52] with 4499 replicates and an overall significance level of 0.05 was used to determine the minimum number of “joinpoints” necessary to fit the data, starting with zero joinpoints and testing whether more joinpoints must be added to the model. A joinpoint is a point of inflection where the linear trend changes. Therefore, joinpoint regression not only evaluates trends but also evaluates variation in trends/slopes. According to the Joinpoint Regression Software algorithmic recommendations, at least four data points between consecutive joinpoints are needed. Therefore, since the data set for this study has eight cycles, it could accommodate up to two joinpoints.

The annual percent change (APC) with the corresponding 95% CI was estimated for each identified trend, by fitting a regression line between the natural logarithm of the prevalence as a dependent variable and year as the independent variable. Furthermore, the following parameter settings were specified in the joinpoint analyses: random errors were assumed to be heteroscedastic (i.e. have non-constant variance using the weighted standard error calculated with Stata 14.0 Software). Moreover, an uncorrelated error structure was used. Regression coefficients were estimated by weighted least squares, and the grid search method was chosen to search for the location of the joinpoints. Significant differences by gender, age groups, rural/ urban area, province or territory, and physical activity levels were detected using a specific comparability test. The test is applied to compare prevalence of CBD trends between two groups using tests of parallelism of time trends. A Bonferroni correction of *p* values was used to consider the multiple pairwise comparisons of the parallelism test, which determines whether the two regression slopes are parallel, allowing different intercepts. The multiple pairwise comparisons for physical activity and ages groups were considered significant if *p* < 0.016 and for province/territories if *p* < 0.0009; in the remaining comparisons, two-sided *p*-values were considered to indicate statistical significance when they were < 0.05.

## Results

### General characteristics

The weighted sample represented a range of 21,415,922 Canadian adults in 2007 up to 22,860,912 Canadian adults in 2014 (Table 2). There was a fairly balanced distribution of both genders. The proportion of respondents aged between 35 and 49 years declined from 35.0% in 2007 to 30.6% in 2014, while the number of respondents aged between 50 and 65 years increased from 30.4% in 2007 to 35.1% in 2014. The range of people living in urban areas was 82.1% in 2012 to 82.8% in 2013. Also, close to 40% of respondents resided in the province of Ontario. Throughout the eight cycles, the prevalence of people classified as inactive fell from 46.8 to 43.5%, while the prevalence of people classified as active increased from 25.4 to 29.5%. General characteristics by year can be found in Table 2.

**Table 2:** Characteristics of study participants. Canadian Community Health Survey (CCHS), 2007–2014

Variable	2007	2008	2009	2010	2011	2012	2013	2014
Gender								
Men	49.9	50.1	50.0	50.0	49.9	49.9	49.9	49.9
Women	50.1	49.9	50.0	50.0	50.1	50.1	50.1	50.1
Age group								
18–34	34.6	34.6	34.1	34.1	34.5	34.3	35.2	34.3
35–49	35.0	34.4	34.0	33.5	32.1	31.4	31.1	30.6
50–65	30.4	31.0	31.9	32.4	33.4	34.3	33.8	35.1
Location								
Urban	82.4	82.2	82.7	82.7	82.7	82.1	82.8	82.2
Rural	17.6	17.8	17.3	17.3	17.3	17.9	17.2	17.8
Province								
Ontario	39.1	39.0	39.0	38.9	38.9	38.9	39.0	38.7
Quebec	23.6	23.4	23.2	23.3	23.3	23.1	23.1	23.3
British Columbia	13.3	13.4	13.5	13.3	13.3	13.3	13.2	13.0
Alberta	10.6	10.7	10.9	11.2	11.2	11.4	11.5	11.9
Manitoba	3.3	3.3	3.3	3.3	3.4	3.4	3.4	3.3
Saskatchewan	2.7	2.8	2.8	2.8	2.8	2.8	2.8	2.9
Nova Scotia	2.8	2.8	2.7	2.7	2.7	2.7	2.7	2.6
New Brunswick	2.3	2.2	2.2	2.2	2.2	2.2	2.1	2.0
Newfoundland and Labrador	1.6	1.6	1.6	1.5	1.5	1.5	1.5	1.5
Prince Edward Island	0.4	0.4	0.4	0.4	0.4	0.4	0.4	0.4
Northern Territories	0.3	0.3	0.3	0.3	0.3	0.3	0.3	0.3
PA level^a^								
Active	25.4	25.7	27.8	27.6	28.9	29.2	30.2	29.5
Moderate	25.3	25.5	25.3	25.8	26.3	25.8	26.0	25.2
Inactive	46.8	46.6	44.7	44.8	43.1	43.4	41.9	43.5
Not-stated	2.6	2.3	2.1	1.8	1.7	1.6	1.8	1.7
CBD Prevalence								
Crude	18.7	22.7	18.9	19.0	18.7	18.1	18.7	17.7
Age-standardized	19.0	23.0	19.2	19.2	18.8	18.2	18.9	17.8
N	21,415,922	21,634,104	21,960,978	22,214,827	22,398,160	22,583,367	22,760,062	22,860,912

^a^PA level: Transportation and leisure physical activity level. Northern Territories: Combined Yukon, Northwest and Nunavut Territories

### Overall and specific CBD prevalence

Both crude and age-standardized CBD prevalence showed that in 2007, nearly 19% reported having CBD, in 2008 the figures peaked at nearly 23%, and by 2014, the CBD prevalence was slightly lower, at about 18%. CBD prevalence was consistently and significantly higher in women, older age groups, rural populations, people living in Nova Scotia and people classified as inactive. The Additional files 1 and 2 show the crude and age-standardized CBD prevalence for each year, respectively.

### Overall prevalence of CBD mapping

**Fig. 1:**
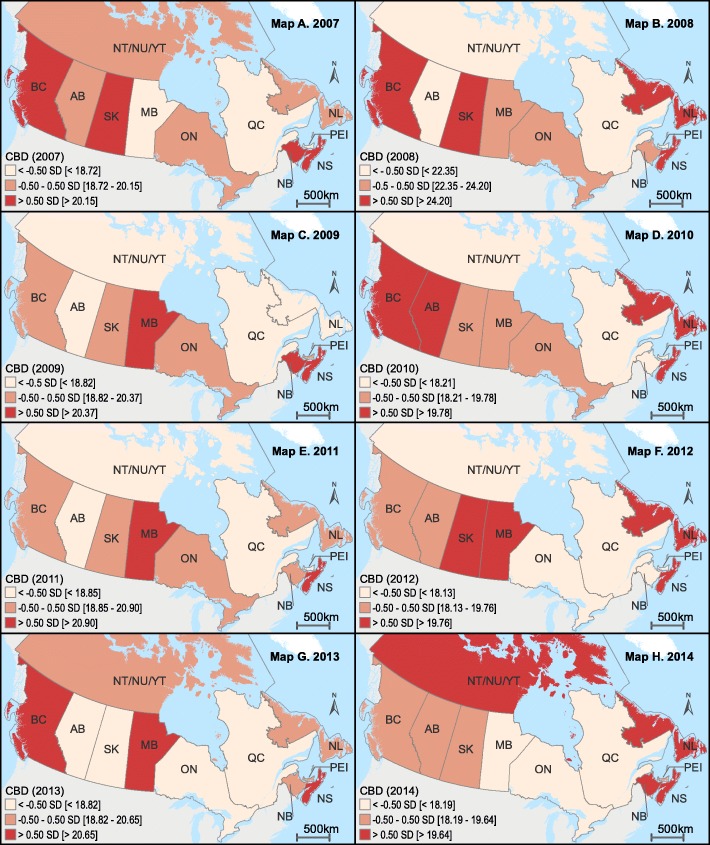
Age-standardized prevalence of CBD among Canadian Provinces and Territories, 2007–2014 (respective maps are A-H). CBD: Chronic Back Disorders; > 0.5 SD; High prevalence; between − 0.50 and 0.50 SD: moderate prevalence; < 0.5 SD low prevalence; ON: Ontario; QC: Quebec; BC: British Columbia; AB: Alberta; MB: Manitoba; SK: Saskatchewan; NS: Nova Scotia; NB: New Brunswick; NL: Newfoundland and Labrador; PEI: Prince Edward Island; NT/NU/YT: Northern Territories, including Yukon, Northwest, and Nunavut Territories

Figure 1 shows the spatial distributions of age-standardized prevalence of CBD across Canadian provinces and territories between 2007 and 2014. Only Nova Scotia and Quebec remained in the same prevalence category in all years. Specifically, Nova Scotia fell into the high CBD prevalence, whereas Quebec fell into the low CBD prevalence category.

### Trends in prevalence of CBD

**Fig. 2:**
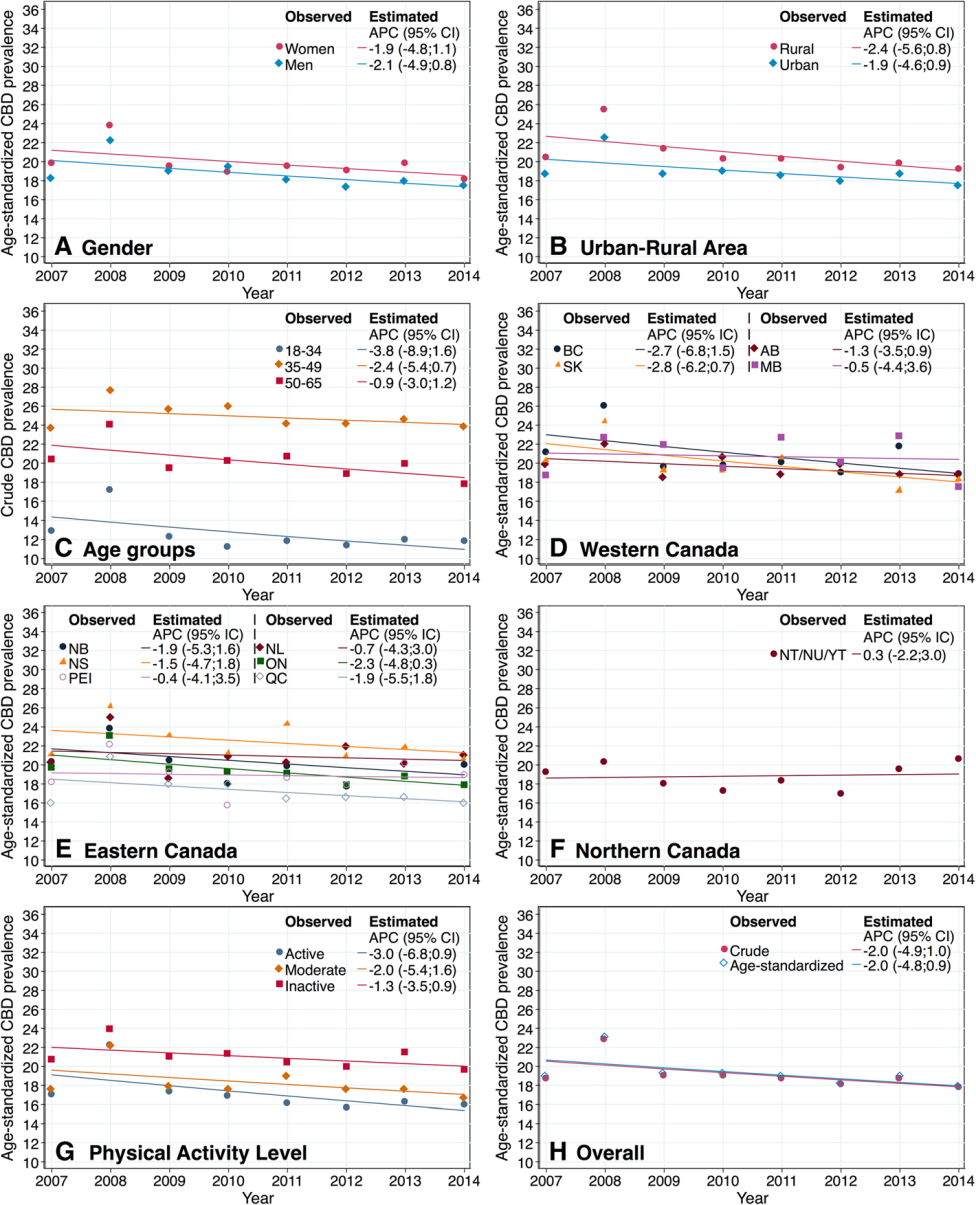
Overall and specific estimates of CBD prevalence in Canada (2007–2014). **a**. Age-standardized CBD prevalence by gender; **b**. Age-standardized CBD prevalence by urban-rural area. **c**. Crude CBD prevalence by age groups. **d**, **e**, **f**. Age-standardized CBD prevalence by provinces and territories. **g** Age-standardized CBD prevalence by physical activity level. **h**. Overall crude and age-standardized CBD prevalence. APC: Annual Percent Change; 95% CI: 95% Confidence Interval; ON: Ontario; QC: Quebec; BC: British Columbia; AB: Alberta; MB: Manitoba; SK: Saskatchewan; NS: Nova Scotia; NB: New Brunswick; NL: Newfoundland and Labrador; PEI: Prince Edward Island; NT/NU/YT: Northern Territories, including Yukon, Northwest, and Nunavut Territories. Trends were not significantly different from zero, apha = 0.05 level

The joinpoint regression analyses showed no statistically significant time trends across all CBD prevalence estimates, meaning that age-standardized prevalence of CBD has been fairly stable from 2007 to 2014 (Fig. 2). Age-standardized CBD prevalence estimates showed only small, non-statistically-significant decreases in annual percent change (APC) by all the different subgroups (negative APC range: − 3.0%; − 0.4%). However, in the Northern Territories the age-standardized CBD prevalence showed a non-statistically-significant increase of 0.3% by year. Similarly, the crude prevalence of CBD remained stable over the study period (See Additional file 2). The pairwise comparison indicated that crude and age-standardized CBD prevalence decreased faster in people classified as physically active compared to those who were inactive (age-standardized APC: physically inactive = − 1.3, physically active − 3.0. *p* < 0.004). There were no other significant differences using the test of parallelism (see supplementary material, Additional file 3, for pairwise test results).

## Discussion

The objective of this study was to examine the trends in the overall prevalence of CBD and specific CBD prevalence by age, gender, residence (rural/urban and province/territory), and physical activity levels among Canadian adults aged 18–65 years. We found that the overall and specific trends in CBD prevalence have been stable from 2007 to 2014 in Canadians aged 18 to 65 years. These findings contrast with the increase in CBD prevalence reported in other high income countries [27–33]. CBD prevalence was consistently higher in women, older age groups, rural populations, and people classified as inactive within each cycle 2007–2014. Trends (Fig. 2e) and maps (Fig. 1) consistently showed that the prevalence of CBD remained relatively higher in Nova Scotia and relatively lower in Quebec in comparison with the remainder of the Canadian provinces and territories.

### Overall trend prevalence

Evidence about the population prevalence of CBD over time in North America is scarce. We found 11 articles studying secular trends in back disorders in Finland [33–35, 37], Britain [27, 28], USA [29], Germany [36], Sweden [32], and Austria [30, 31]. All studies included information collected before 2007, and only one study focused on chronic problems [29]. Seven of the studies conducted in Finland [34, 35, 37], Austria [30, 31], Britain [28], and Germany [36] reported prevalence estimates of back disorders greater than 20% for at least one study period. While the remaining four studies from Finland [33], Britain [27], Sweden [32], and USA [29] showed a prevalence lower or equal to 20%. The lower CBD prevalence found in the present study could be partially explained by the definition of the outcome, which includes back problems that have lasted at least six months, while the studies with greater prevalence did not explicitly define the duration of back pain. Thus, the back pain definition in the previous studies may include those who have acute back problems lasting less than six months duration, leading to higher prevalence rates.

Stable chronic pain prevalence rates were also found by Reitsma et al. [53] using two Canadian surveys, the National Population Health Survey (cycles 1994/5, 1996/7, 1998/9), and the CCHS (from 2000/1 to 2007/8). The former survey included people aged 25 years and older, and the latter included people aged 20 years and older. Chronic pain was defined as a negative response to the question: “Are you usually free of pain or discomfort?”, which would likely include people with CBD [53]. Reitsma et al. results indicated that the overall temporal trends were not significant. The initial prevalence was 18.9%, the final prevalence was 18.5%, and the minimum prevalence was 15.1% in 1996/97 [53]. They also affirmed that studies using a specific timeframe to define chronic pain such as three or six months were more likely to find greater chronic pain prevalence compared to using an unspecified timeframe like “usual” pain [53].

Our results also concur with those reported by Leino et al. [35]. In their study of back problems from 1979 to 1992 in Germany, Leino et al. reported back-pain prevalence close to 30%, but did not find changes between 1985 and 1992. Leino et al. stated that the stable prevalence could be explained by changes in the societal judgment of good health and functional capacity, and attitudes towards pain. Thus, although people reported more acute musculoskeletal problems, the improvement of the health care system has prevented the development of more severe chronic conditions after the onset of back pain. While this trade-off phenomenon might have been at work in 1980s and 1990s Germany, a similar tradeoff may be in effect during the later period investigated in this current work, where a stable CBD prevalence could be the result of following the evolving guidelines for back pain management [54]. The guidelines recommended staying active instead of bed rest in order to reduce fear-avoidance beliefs and other negative psychological consequences. Following these guidelines could reduce the number of people progressing from acute to chronic back pain, thus those suffering during at least six months remain stable under the assumption of a stable incidence [54].

Another possible explanation of fairly stable CBD prevalence is that in our study CBD was measured using consistent, comparable questions with similar CCHS methods. The five [27, 30–32, 35] out of the eight population-based studies showing an increased trend in CBD [27–33, 35] reported methodological changes over time involving variable: response options [35]; mode of data collection [27]; sampling process [32]; and questions asked to ascertain CBD [30, 31]. Modifications in the questions and survey methods may mask real trends in CBD due to the inability to determine whether observed trends are due to real changes in population morbidity or due to changes in research methodology.

### Specific trend prevalence

#### Gender and age

Our findings relating to a higher CBD prevalence in women and older people concurred with other studies. Among studies evaluating trends in CDB, Leijon et al. [32], Harkness et al. [27], and Freburger et al. [29] found that CBD prevalence rates were significantly greater in women than men across time. In general, trends in CBD prevalence in both men and women followed the overall trend except in two studies [32, 34]. Leijon et al. [32] found that the increasing trend was statistically significant among women but not among men, and Heistaro et al. [34] revealed that after controlling for age, the declining trend was statistically significant among men but not among women. On the other hand, Heistaro et al. [34], Palmer et al. [28], Harkness et al. [27] and Großschadl et al. [30, 31] found greater CBD prevalence in older (mainly over 55 years) people. In our study, we found that the slope of the younger group was greater in the 18–34 years group compared to the 50–65 years group, meaning that the CBD prevalence declined more slowly in the 50–65 years group. A similar pattern was found by Heistaro et al. [34], whose time graphs showed that the significant decreasing trend was more noticeable in younger people (30–39 years and 40–49 years) than older people (50–59 years).

#### Geographical variations

In this study, rural dwellers had a higher CBD prevalence over time in comparison to urban dwellers. In addition, trends and maps consistently showed that the prevalence of CBD remained relatively high in the province of Nova Scotia and relatively low in Quebec. The high CBD prevalence in the province of Nova Scotia could be due partly to the greater percentage (34%) of rural population in that province, which is higher than the proportion of rural population in Quebec (19%) [55]. The higher CBD prevalence in the Yukon, Northwest and Nunavut Territories and rural settings may be related to challenges in accessing health care services. People living in rural or remote places have more difficulties to get health care services compared with those living in urban settings [56]. It is probable that in remote and rural places, people with an acute back problem are less likely to receive adequate or any health care advice or treatment, which may lead to a chronic condition. In addition, people living in rural and remote places are involved in different industries and working conditions (e.g. agriculture, mining), and thus they could be exposed to different risk factors for CBD [57]. Furthermore, there is a high proportion of Indigenous people living in the Canadian Northern territories [58] and Indigenous people have a higher reported prevalence of CBD in comparison to non-Indigenous populations [57].

#### Physical activity

We found that people classified as inactive had a higher CBD prevalence over the eight cycles. In addition, we found a slope difference between inactive and active people, indicating that the CBD prevalence decreased faster in active people than their physically inactive counterparts. Differences in CBD prevalence trends by the level of leisure time physical activity were found by Heistaro et al. [34]. Their time graphs showed that the significant decreasing trend was more noticeable in men engaged in high physical activity than men engaged in moderate or low physical activity. In contrast, women engaged in high physical activity exhibited a rising trend in back pain prevalence. These findings, however, are not completely comparable with our results because the questionnaire used was different, and we included both transportation and leisure physical activity in the analysis.

There are some proposed mechanisms that explain how physical activity could decrease the likelihood of having CBD. First, being physically active may strengthen back muscles and increase trunk flexibility that can provide the stability and range of motion needed in functional activities, and thus the risk of back injury will be reduced [59]. Second, physical activity may increase circulation of the blood to the back muscles, joints, and intervertebral fibrocartilage, reducing damage and stiffness that can result in back pain [60]. Third, people who regularly exercise can complete daily tasks with less effort, thus decreasing fatigue, and maintain muscular strength and endurance; which may ultimately reduce the likelihood of overload from daily tasks later in life [61, 62]. Moreover, regular physical activity reduce serotonin transporter expression, increases serotonin levels, and increases endogenous opioids in central inhibitory pathways; decreasing the perception of back pain [63].

### Strengths and limitations

One strength of our study was the use of eight fully comparable CCHS cycles. The same CBD question employed in this study allowed an exploration of time trends in a large and representative sample of Canadians aged 18 to 65 years. In addition, we used a joinpoint regression analysis that has never been utilized in CBD trends research; this flexible statistical method is useful for the assessment of changes of the outcome (CBD prevalence) as a function of the independent variable (year, from 2007 to 2014) in general and by groups. Joinpoint regression analysis was initially used to determine the change points and trend pattern of cancer rates [52]. However, to our knowledge, this is the first time it is being used in CBD trends research.

There are some limitations in this study to consider when interpreting our findings. The first limitation is the CBD classification. As there was not a body diagram alongside the CBD question, the CBD category might contain those who have only upper back pain, and those with concomitant neck, upper and lower back pain. Similarly, although the CCHS’s CBD question asked for back problems excluding fibromyalgia and arthritis, people with concomitant fibromyalgia and arthritis may also have CBD [16]. The prevalence of arthritis and fibromyalgia was around 16% [64] and 2% [65] in Canada (2014), respectively, this implies an underestimation of the CBD prevalence up to 16% on an 18% prevalence of CBD in the same year. Also, interpreting the CCHS data relies on the assumption that respondents have correctly interpreted the questions. For example, some vocabulary such as fibromyalgia/arthritis might be unfamiliar, though we anticipate that those who received a diagnosis will have heard the terms from their care providers. Furthermore, self-report questionnaires introduce possible biases, including recall error [66]. In addition, the method of interview could influence the participants’ response. For example, St-Pierre found that in the 2003 CCHS, people interviewed in-person reported being more inactive (42.3%) than people interviewed by telephone (34.4%) [67]. A further limitation is that the generalizability of the results to the complete Canadian population or the European population may be restricted due to contextual factors and the exclusion of people living on reserves and other aboriginal settlements, residents of institutional facilities, members of the Canadian Forces, and residents of certain remote regions.

### Future work, implications and applications

The great variety of questions used to determine CBD prevalence makes comparisons among studies difficult. Thus, it is important for researchers to report the features of the CBD questions used as well as disclose any modifications in the methodology to allow for the appropriate interpretation of their results. Cross-sectional surveys do not allow identification of causal relationships. Consequently, next steps would incorporate analysis of longitudinal data that include repeated measurements of CBD prevalence. This study contributes to the understanding of the epidemiology of CBD using relatively recent data. In addition, we believe that ongoing monitoring of CBD over time could be used to evaluate the effectiveness of population-based prevention programs and management approaches among adults suffering from back disorders.

## Conclusions

CBD represents a public health problem in Canada impacting one in five adults. However, despite prevention efforts such as the guide on the prevention of musculoskeletal injuries, public health campaigns, printed materials, and mobile applications, prevalence rates have been stable from 2007 to 2014. Furthermore, the prevalence rates of CBD among all specific subgroups also have been steady, with the highest prevalence among women, rural residence, physically inactive and older people as well as in people living in Nova Scotia, in comparison to other Canadian provinces and territories. Our findings also indicate that CBD prevalence is lower and decreasing more rapidly in younger and physically active people compared to older and inactive people respectively. Therefore, we recommend that preventive strategies should be targeted for women, older, inactive people, and people living in rural and remote areas.

## Additional files

Additional file 1: Overall and specific age-standardized prevalence of CBD and joinpoint regression analysis. Canadian Community Health Survey, 2007–2014. (DOCX 26 kb)

Additional file 2: Overall and specific crude prevalence of CBD and joinpoint regression analysis. Canadian Community Health Survey, 2007–2014. (DOCX 28 kb)

Additional file 3: Pairwise comparison of trend data using the test of parallelism. (DOCX 24 kb)

## Data Availability

The data that support the findings of this study are available from Statistics Canada (Government of Canada) but restrictions apply to the availability of the raw data, which were used under permission for the current study, and so are not publicly available. Data are however available from the authors upon reasonable request and with permission of The Research Data Center Program.

## Abbreviations

95% CI: 95% confidence intervals
AB: Alberta
APC: annual percent change
BC: British Columbia
BRR: Balanced repeated replications
CBD: Chronic back disorders
CCHS: Canadian Community Health Survey
MB: Manitoba
NB: New Brunswick
NL: Newfoundland and Labrador
NS: Nova Scotia
NT/NU/YT: Northern Territories, including Yukon, Northwest, and Nunavut Territories
ON: Ontario
PEI: Prince Edward Island
QC: Quebec
RDD: Random digit dialing
SD: Standard deviation
SK: Saskatchewan
