# Supplementary material for: Stable prevalence of chronic back disorders across gender, age, residence, and physical activity in Canadian adults from 2007 to 2014
Source: BMC Public Health. 2019 Aug 15;19:1121. doi: 10.1186/s12889-019-7395-8 (PMC6694571; doi:10.1186/s12889-019-7395-8)
Supplement: Supplementary file 1 — Overall and specific age-standardized prevalence of CBD and joinpoint regression analysis. Canadian Community Health Survey, 2007–2014. (DOCX 26 kb) [file 12889_2019_7395_MOESM1_ESM.docx]

**Additional File 1. Age-standardized prevalence of CBD and joinpoint regression analysis. Canadian Community Health Survey, 2007 - 2014**

| **Type of prevalence** | **2007** | | **2008** | | **2009** | | **2010** | | **2011** | | **2012** | | **2013** | | **2014** | | **Joinpoint regression analysis** | | |
| --- | --- | --- | --- | --- | --- | --- | --- | --- | --- | --- | --- | --- | --- | --- | --- | --- | --- | --- | --- |
|  | **P** | **95% CI** | **P** | **95% CI** | **P** | **95% CI** | **P** | **95% CI** | **P** | **95% CI** | **P** | **95% CI** | **P** | **95% CI** | **P** | **95% CI** | **APC** | **95% CI** | **p-value** |
| **Specific by** |  |  |  |  |  |  |  |  |  |  |  |  |  |  |  |  |  |  |  |
| Gender |  |  |  |  |  |  |  |  |  |  |  |  |  |  |  |  |  |  |  |
| Women | 19.7 | 19.0;20.5 | 23.8 | 22.9;24.7 | 19.4 | 18.6;20.3 | 19.0 | 18.1;19.8 | 19.6 | 18.7;20.4 | 19.0 | 18.1;20.0 | 19.8 | 18.9;20.8 | 18.2 | 17.3;19.1 | -1.9 | -4.8;1.1 | 0.173 |
| Men | 18.2 | 17.4;19.0 | 22.1 | 21.3;23.0 | 18.9 | 18.0;19.8 | 19.4 | 18.4;20.3 | 18.1 | 17.2;19.0 | 17.3 | 16.3;18.2 | 17.9 | 16.9;18.9 | 17.4 | 16.5;18.3 | -2.1 | -4.9;0.8 | 0.128 |
| Area |  |  |  |  |  |  |  |  |  |  |  |  |  |  |  |  |  |  |  |
| Urban | 18.6 | 18.0;19.2 | 22.5 | 21.8;23.2 | 18.7 | 18.0;19.4 | 19.0 | 18.2;19.7 | 18.5 | 17.8;19.3 | 17.9 | 17.1;18.7 | 18.7 | 17.9;19.4 | 17.4 | 16.7;18.2 | -1.9 | -4.6;0.9 | 0.150 |
| Rural | 20.4 | 19.4;21.4 | 25.4 | 24.2;26.5 | 21.3 | 20.2;22.5 | 20.3 | 19.1;21.5 | 20.2 | 19.0;21.4 | 19.4 | 18.1;20.6 | 19.8 | 18.6;21.1 | 19.3 | 18.0;20.5 | -2.4 | -5.6;0.8 | 0.117 |
| Province |  |  |  |  |  |  |  |  |  |  |  |  |  |  |  |  |  |  |  |
| Ontario | 19.6 | 18.7;20.5 | 23.1 | 22.0;24.1 | 19.5 | 18.5;20.6 | 19.3 | 18.2;20.3 | 19.1 | 18.0;20.2 | 17.9 | 16.7;19.0 | 18.8 | 17.6;20.0 | 17.8 | 16.8;18.9 | -2.3 | -4.8;0.3 | 0.070 |
| Quebec | 15.9 | 14.8;17.0 | 20.8 | 19.5;22.1 | 17.9 | 16.6;19.1 | 17.9 | 16.5;19.3 | 16.3 | 15.0;17.7 | 16.5 | 15.1;17.8 | 16.5 | 15.2;17.8 | 16.0 | 14.5;17.4 | -1.9 | -5.5;1.8 | 0.246 |
| British Columbia | 21.0 | 19.5;22.5 | 26.0 | 24.4;27.6 | 19.6 | 18.0;21.2 | 19.8 | 18.1;21.5 | 20.1 | 18.3;21.9 | 19.0 | 17.1;20.9 | 21.7 | 19.4;23.9 | 18.9 | 17.1;20.6 | -2.7 | -6.8;1.5 | 0.160 |
| Alberta | 19.7 | 18.0;21.5 | 21.9 | 20.0;23.8 | 18.4 | 16.6;20.2 | 20.6 | 18.4;22.8 | 18.7 | 16.8;20.6 | 19.8 | 17.5;22.1 | 18.7 | 16.6;20.9 | 18.8 | 16.7;20.8 | -1.3 | -3.5;0.9 | 0.203 |
| Manitoba | 18.6 | 16.5;20.8 | 22.7 | 20.4;25.0 | 21.9 | 18.9;24.9 | 19.4 | 16.5;22.3 | 22.6 | 19.3;25.9 | 20.0 | 16.8;23.2 | 22.8 | 19.9;25.7 | 17.4 | 15.0;19.9 | -0.5 | -4.4;3.6 | 0.787 |
| Saskatchewan | 20.2 | 18.1;22.3 | 24.3 | 22.1;26.6 | 19.2 | 16.8;21.6 | 19.1 | 16.7;21.5 | 20.5 | 17.8;23.2 | 19.9 | 17.6;22.3 | 17.1 | 14.6;19.6 | 18.2 | 15.9;20.6 | -2.8 | -6.2;0.7 | 0.092 |
| Nova Scotia | 21.1 | 18.7;23.5 | 26.0 | 23.4;28.7 | 23.1 | 20.2;25.9 | 21.2 | 18.4;23.9 | 24.3 | 21.3;27.3 | 21.0 | 17.9;24.0 | 21.7 | 18.8;24.7 | 20.6 | 18.0;23.3 | -1.5 | -4.7;1.8 | 0.310 |
| New Brunswick | 20.3 | 18.1;22.5 | 23.8 | 21.7;26.0 | 20.4 | 17.7;23.1 | 18.0 | 15.5;20.4 | 19.9 | 17.1;22.6 | 17.7 | 15.0;20.5 | 20.2 | 17.5;22.9 | 20.0 | 17.3;22.7 | -1.9 | -5.3;1.6 | 0.232 |
| Newfoundland  and Labrador | 20.1 | 17.6;22.6 | 24.9 | 21.9;28.0 | 18.4 | 15.7;21.2 | 20.7 | 17.7;23.7 | 20.2 | 17.1;23.4 | 21.9 | 18.2;25.5 | 20.1 | 17.0;23.2 | 20.9 | 17.8;24.0 | -0.7 | -4.3;3.0 | 0.668 |
| Prince Edward  Island | 18.1 | 15.2;21.0 | 22.2 | 18.4;26.0 | 19.2 | 14.9;23.4 | 15.7 | 12.1;19.3 | 18.6 | 14.2;22.9 | 17.8 | 13.8;21.9 | 20.1 | 15.9;24.3 | 18.9 | 14.9;22.9 | -0.4 | -4.1;3.5 | 0.813 |
| Northern Territories | 19.1 | 16.4;21.9 | 20.2 | 17.0;23.5 | 17.9 | 15.0;20.9 | 17.3 | 14.3;20.2 | 18.4 | 15.3;21.5 | 17.0 | 14.0;20.0 | 19.5 | 17.0;21.9 | 20.6 | 17.3;23.9 | 0.3 | -2.2;3.0 | 0.305 |
| PA level |  |  |  |  |  |  |  |  |  |  |  |  |  |  |  |  |  |  |  |
| Active | 17.1 | 16.1;18.0 | 22.1 | 20.9;23.3 | 17.3 | 16.2;18.5 | 16.8 | 15.6;18.1 | 16.0 | 14.9;17.2 | 15.6 | 14.4;16.7 | 16.2 | 15.0;17.3 | 15.9 | 14.8;17.1 | -3.0 | -6.8;0.9 | 0.108 |
| Moderate | 17.6 | 16.6;18.6 | 22.1 | 20.9;23.3 | 17.8 | 16.6;19.0 | 17.6 | 16.4;18.8 | 18.9 | 17.7;20.1 | 17.6 | 16.3;18.9 | 17.6 | 16.3;18.8 | 16.6 | 15.3;17.8 | -2.0 | -5.4;1.6 | 0.219 |
| Inactive | 20.7 | 19.9;21.5 | 23.9 | 22.9;24.8 | 21.0 | 20.1;21.9 | 21.2 | 20.2;22.3 | 20.4 | 19.3;21.4 | 19.9 | 18.9;21.0 | 21.4 | 20.2;22.5 | 19.5 | 18.5;20.5 | -1.3 | -3.5;0.9 | 0.193 |
| **Overall** |  |  |  |  |  |  |  |  |  |  |  |  |  |  |  |  |  |  |  |
| Age-standardized | 18.9 | 18.4;19.5 | 22.9 | 22.3;23.6 | 19.2 | 18.6;19.7 | 19.2 | 18.5;19.8 | 18.8 | 18.2;19.5 | 18.2 | 17.5;18.8 | 18.9 | 18.2;19.5 | 17.8 | 17.2;18.4 | -2.0 | -4.8;0.9 | 0.140 |

P= Prevalence. 95% CI= 95% Confidence Interval. APC=Annual Percent Change. Northern Territories: Combined Yukon, Northwest and Nunavut Territories. PA level: Transportation and leisure physical activity level.
