# Supplementary material for: Stable prevalence of chronic back disorders across gender, age, residence, and physical activity in Canadian adults from 2007 to 2014
Source: BMC Public Health. 2019 Aug 15;19:1121. doi: 10.1186/s12889-019-7395-8 (PMC6694571; doi:10.1186/s12889-019-7395-8)
Supplement: Supplementary file 2 — Overall and specific crude prevalence of CBD and joinpoint regression analysis. Canadian Community Health Survey, 2007–2014. (DOCX 28 kb) [file 12889_2019_7395_MOESM2_ESM.docx]

**Additional File 2. Crude prevalence of CBD and joinpoint regression analysis. Canadian Community Health Survey, 2007 - 2014**

| **Type of prevalence** | **2007** | | **2008** | | **2009** | | **2010** | | **2011** | | **2012** | | **2013** | | **2014** | | **Joinpoint regression analysis** | | |
| --- | --- | --- | --- | --- | --- | --- | --- | --- | --- | --- | --- | --- | --- | --- | --- | --- | --- | --- | --- |
|  | **P** | **95% CI** | **P** | **95% CI** | **P** | **95% CI** | **P** | **95% CI** | **P** | **95% CI** | **P** | **95% CI** | **P** | **95% CI** | **P** | **95% CI** | **APC** | **95% CI** | **p-value** |
| **Specific by** |  |  |  |  |  |  |  |  |  |  |  |  |  |  |  |  |  |  |  |
| Gender |  |  |  |  |  |  |  |  |  |  |  |  |  |  |  |  |  |  |  |
| Women | 19.6 | 18.8;20.3 | 23.6 | 22.8;24.5 | 19.2 | 18.4;20.1 | 18.8 | 17.9;19.7 | 19.5 | 18.6;20.4 | 19.0 | 18.1;20.0 | 19.7 | 18.8;20.7 | 18.2 | 17.3;19.1 | -1.9 | -4.9;1.2 | 0.185 |
| Men | 17.9 | 17.1;18.7 | 21.9 | 21.0;22.7 | 18.6 | 17.8;19.5 | 19.2 | 18.3;20.2 | 17.9 | 17.0;18.9 | 17.2 | 16.2;18.1 | 17.6 | 16.7;18.6 | 17.3 | 16.4;18.3 | -2.1 | -4.9;0.9 | 0.135 |
| Age groups (years) |  |  |  |  |  |  |  |  |  |  |  |  |  |  |  |  |  |  |  |
| 18-34 | 12.9 | 12.1;13.7 | 17.2 | 16.3;18.1 | 12.2 | 11.4;13.0 | 11.2 | 10.5;12.0 | 11.8 | 10.9;12.7 | 11.4 | 10.5;12.3 | 11.9 | 11.1;12.9 | 11.7 | 10.8;12.7 | -3.8 | -8.9;1.6 | 0.136 |
| 35-49 | 20.3 | 19.4;21.3 | 24.0 | 22.9;25.1 | 19.5 | 18.4;20.6 | 20.2 | 18.9;21.5 | 20.6 | 19.2;22.1 | 18.8 | 17.6;20.1 | 20.0 | 18.6;21.4 | 17.7 | 16.5;19.0 | -2.4 | -5.4;0.7 | 0.106 |
| 50-65 | 23.6 | 22.6;24.6 | 27.6 | 26.5;28.7 | 25.6 | 24.4;26.8 | 25.9 | 24.6;27.2 | 24.0 | 22.9;25.2 | 24.1 | 22.8;25.4 | 24.6 | 23.3;25.8 | 23.7 | 22.6;24.9 | -0.9 | -3.0;1.2 | 0.330 |
| Area |  |  |  |  |  |  |  |  |  |  |  |  |  |  |  |  |  |  |  |
| Urban | 18.3 | 17.7;18.9 | 22.1 | 21.4;22.8 | 18.3 | 17.6;19.0 | 18.5 | 17.8;19.3 | 18.2 | 17.5;19.0 | 17.7 | 16.9;18.4 | 18.3 | 17.5;19.1 | 17.2 | 16.5;18.0 | -1.9 | -4.8;1.0 | 0.157 |
| Rural | 20.9 | 19.8;21.9 | 25.8 | 24.7;26.8 | 22.0 | 20.8;23.3 | 21.2 | 20.0;22.3 | 21.0 | 19.8;22.3 | 20.0 | 18.8;21.4 | 20.7 | 19.4;21.9 | 20.3 | 19.1;21.6 | -2.2 | -5.2;1.0 | 0.140 |
| Province |  |  |  |  |  |  |  |  |  |  |  |  |  |  |  |  |  |  |  |
| Ontario | 19.4 | 18.6;20.2 | 22.8 | 21.8;23.8 | 19.2 | 18.2;20.2 | 19.0 | 17.9;20.1 | 18.9 | 17.8;20.1 | 17.8 | 16.7;19.0 | 18.6 | 17.4;19.9 | 17.8 | 16.7;18.9 | -2.2 | -4.8;0.4 | 0.087 |
| Quebec | 15.9 | 14.8;17.1 | 20.8 | 19.5;22.1 | 17.8 | 16.6;19.2 | 17.9 | 16.5;19.5 | 16.4 | 15.1;17.8 | 16.5 | 15.2;17.8 | 16.5 | 15.2;17.9 | 16.0 | 14.5;17.5 | -2.0 | -5.6;1.7 | 0.235 |
| British Columbia | 20.8 | 19.4;22.2 | 25.8 | 24.3;27.4 | 19.5 | 17.9;21.1 | 19.7 | 18.0;21.5 | 19.9 | 18.1;21.8 | 18.9 | 17.1;20.8 | 21.7 | 19.4;24.2 | 18.9 | 17.2;20.8 | -2.6 | -6.7;1.7 | 0.191 |
| Alberta | 18.9 | 17.3;20.7 | 21.4 | 19.7;23.2 | 17.5 | 15.9;19.2 | 19.7 | 17.8;21.8 | 18.0 | 16.2;19.9 | 19.2 | 16.9;21.8 | 18.1 | 16.0;20.4 | 18.2 | 16.3;20.3 | -1.2 | -3.8;1.5 | 0.303 |
| Manitoba | 18.0 | 16.1;20.1 | 22.7 | 20.4;25.3 | 21.9 | 19.1;25.0 | 19.1 | 16.5;22.0 | 22.6 | 19.2;26.3 | 19.8 | 17.0;22.9 | 22.4 | 19.5;25.7 | 17.4 | 15.1;20.0 | -0.3 | -4.6;4.1 | 0.853 |
| Saskatchewan | 19.7 | 17.8;21.8 | 23.9 | 21.8;26.2 | 18.6 | 16.3;21.2 | 18.6 | 16.2;21.2 | 20.2 | 17.6;23.1 | 19.4 | 17.1;22.0 | 16.5 | 14.3;19.0 | 17.9 | 15.6;20.3 | -2.9 | -6.4;0.7 | 0.095 |
| Nova Scotia | 21.0 | 18.8;23.3 | 26.3 | 23.7;29.0 | 23.5 | 20.8;26.3 | 21.5 | 18.8;24.5 | 24.7 | 21.9;27.7 | 21.0 | 17.8;24.7 | 22.2 | 19.5;25.1 | 21.1 | 18.7;23.6 | -1.2 | -4.4;2.2 | 0.422 |
| New Brunswick | 20.5 | 18.3;22.8 | 24.1 | 22.0;26.4 | 20.5 | 17.8;23.5 | 18.3 | 15.9;21.0 | 20.2 | 17.9;22.8 | 18.1 | 15.4;21.3 | 20.7 | 18.2;23.5 | 20.8 | 17.9;24.0 | -1.6 | -5.0;2.0 | 0.320 |
| Newfoundland  and Labrador | 20.3 | 18.0;22.9 | 24.7 | 21.8;27.8 | 19.2 | 16.4;22.3 | 21.5 | 18.7;24.6 | 20.5 | 17.6;23.8 | 22.4 | 19.0;26.1 | 20.3 | 17.3;23.7 | 21.7 | 18.8;24.9 | -0.2 | -3.3;3.0 | 0.864 |
| Prince Edward  Island | 18.0 | 15.1;21.2 | 22.4 | 18.8;26.4 | 19.9 | 15.7;24.9 | 16.0 | 12.8;19.7 | 18.9 | 15.2;23.2 | 18.6 | 15.0;22.9 | 20.0 | 16.4;24.2 | 19.4 | 15.9;23.4 | -0.1 | -3.9;3.8 | 0.929 |
| Northern Territories | 18.3 | 15.9;21.1 | 19.2 | 16.2;22.6 | 17.3 | 14.5;20.5 | 16.5 | 14.0;19.3 | 17.3 | 14.1;21.0 | 16.3 | 13.5;19.6 | 19.1 | 16.7;21.6 | 19.8 | 16.5;23.6 | 0.6 | -2.1;3.3 | 0.640 |
| PA level |  |  |  |  |  |  |  |  |  |  |  |  |  |  |  |  |  |  |  |
| Active | 16.2 | 15.3;17.2 | 21.3 | 20.2;22.5 | 16.5 | 15.4;17.6 | 15.9 | 14.8;17.1 | 15.1 | 14.1;16.3 | 15.0 | 13.9;16.1 | 15.4 | 14.3;16.6 | 15.5 | 14.5;16.6 | -3.0 | -7.1;1.2 | 0.132 |
| Moderate | 17.5 | 16.4;18.6 | 21.9 | 20.7;23.2 | 17.6 | 16.4;18.9 | 17.4 | 16.2;18.7 | 19.0 | 17.8;20.3 | 17.7 | 16.5;19.1 | 17.5 | 16.3;18.7 | 16.5 | 15.3;17.8 | -1.8 | -5.2;1.6 | 0.239 |
| Inactive | 20.8 | 20.0;21.6 | 24.1 | 23.1;25.0 | 21.3 | 20.4;22.2 | 21.8 | 20.7;22.8 | 20.9 | 19.8;22.0 | 20.4 | 19.2;21.6 | 21.9 | 20.7;23.1 | 20.0 | 18.9;21.1 | -1.0 | -3.3;1.3 | 0.329 |
| **Overall** |  |  |  |  |  |  |  |  |  |  |  |  |  |  |  |  |  |  |  |
| Crude | 18.7 | 18.2;19.3 | 22.8 | 22.2;23.3 | 18.9 | 18.4;19.5 | 19.0 | 18.3;19.6 | 18.7 | 18.0;19.4 | 18.1 | 17.4;18.8 | 18.7 | 18.0;19.4 | 17.8 | 17.1;18.4 | -2.0 | -4.9;1.0 | 0.157 |

P= Prevalence. 95% CI= 95% Confidence Interval. APC=Annual Percent Change. Northern Territories: Combined Yukon, Northwest and Nunavut Territories. PA level: Transportation and leisure physical activity level
