# Supplementary material for: Stable prevalence of chronic back disorders across gender, age, residence, and physical activity in Canadian adults from 2007 to 2014
Source: BMC Public Health. 2019 Aug 15;19:1121. doi: 10.1186/s12889-019-7395-8 (PMC6694571; doi:10.1186/s12889-019-7395-8)
Supplement: Supplementary file 3 — Pairwise comparison of trend data using the test of parallelism. (DOCX 24 kb) [file 12889_2019_7395_MOESM3_ESM.docx]

**Additional File 3. Pairwise comparison of trend data using the test of parallelism.**

| **Trend 1** | **Trend 2** | **Critical p-value**  **Bonferroni Correction** | **Test of parallelism, p-value** | |
| --- | --- | --- | --- | --- |
|  |  |  | **Crude Prevalence** | **Age-Standardized**  **Prevalence** |
| **Age Group** |  |  |  |  |
| 18-34 | 35-49 | 0.0169 | 0.123 | NA |
| 18-34 | 50-65 | 0.0169 | 0.030 | NA |
| 35-49 | 50-65 | 0.0169 | 0.150 | NA |
| **Gender** |  |  |  |  |
| Men | Women | 0.0500 | 0.670 | 0.657 |
| **Urban/rural area** |  |  |  |  |
| Rural | Urban | 0.0500 | 0.587 | 0.275 |
| **Provinces and territories** |  |  |  |  |
| Alberta | British Columbia | 0.0009 | 0.290 | 0.317 |
| Alberta | Manitoba | 0.0009 | 0.605 | 0.629 |
| Alberta | New Brunswick | 0.0009 | 0.614 | 0.365 |
| Alberta | Newfoundland and Labrador | 0.0009 | 0.047 | 0.202 |
| Alberta | Northern Territories | 0.0009 | 0.203 | 0.193 |
| Alberta | Nova Scotia | 0.0009 | 0.925 | 0.810 |
| Alberta | Ontario | 0.0009 | 0.414 | 0.441 |
| Alberta | Prince Edward Island | 0.0009 | 0.202 | 0.241 |
| Alberta | Quebec | 0.0009 | 0.575 | 0.622 |
| Alberta | Saskatchewan | 0.0009 | 0.053 | 0.088 |
| British Columbia | Manitoba | 0.0009 | 0.141 | 0.162 |
| British Columbia | New Brunswick | 0.0009 | 0.472 | 0.515 |
| British Columbia | Newfoundland and Labrador | 0.0009 | 0.224 | 0.224 |
| British Columbia | Northern Territories | 0.0009 | 0.196 | 0.226 |
| British Columbia | Nova Scotia | 0.0009 | 0.279 | 0.368 |
| British Columbia | Ontario | 0.0009 | 0.436 | 0.357 |
| British Columbia | Prince Edward Island | 0.0009 | 0.237 | 0.244 |
| British Columbia | Quebec | 0.0009 | 0.543 | 0.469 |
| British Columbia | Saskatchewan | 0.0009 | 0.855 | 0.948 |
| Manitoba | New Brunswick | 0.0009 | 0.473 | 0.445 |
| Manitoba | Newfoundland and Labrador | 0.0009 | 0.952 | 0.924 |
| Manitoba | Northern Territories | 0.0009 | 0.646 | 0.746 |
| Manitoba | Nova Scotia | 0.0009 | 0.438 | 0.424 |
| Manitoba | Ontario | 0.0009 | 0.161 | 0.202 |
| Manitoba | Prince Edward Island | 0.0009 | 0.859 | 0.948 |
| Manitoba | Quebec | 0.0009 | 0.290 | 0.404 |
| Manitoba | Saskatchewan | 0.0009 | 0.224 | 0.245 |
| New Brunswick | Newfoundland and Labrador | 0.0009 | 0.087 | 0.075 |
| New Brunswick | Northern Territories | 0.0009 | 0.066 | 0.078 |
| New Brunswick | Nova Scotia | 0.0009 | 0.624 | 0.600 |
| New Brunswick | Ontario | 0.0009 | 0.649 | 0.763 |
| New Brunswick | Prince Edward Island | 0.0009 | 0.039 | 0.026 |
| New Brunswick | Quebec | 0.0009 | 0.802 | 0.985 |
| New Brunswick | Saskatchewan | 0.0009 | 0.348 | 0.424 |
| Newfoundland and Labrador | Northern Territories | 0.0009 | 0.585 | 0.497 |
| Newfoundland and Labrador | Nova Scotia | 0.0009 | 0.433 | 0.386 |
| Newfoundland and Labrador | Ontario | 0.0009 | 0.187 | 0.229 |
| Newfoundland and Labrador | Prince Edward Island | 0.0009 | 0.916 | 0.751 |
| Newfoundland and Labrador | Quebec | 0.0009 | 0.260 | 0.441 |
| Newfoundland and Labrador | Saskatchewan | 0.0009 | 0.032 | 0.023 |
| Northern Territories | Nova Scotia | 0.0009 | 0.410 | 0.351 |
| Northern Territories | Ontario | 0.0009 | 0.269 | 0.206 |
| Northern Territories | Prince Edward Island | 0.0009 | 0.613 | 0.592 |
| Northern Territories | Quebec | 0.0009 | 0.377 | 0.407 |
| Northern Territories | Saskatchewan | 0.0009 | 0.197 | 0.195 |
| Nova Scotia | Ontario | 0.0009 | 0.244 | 0.509 |
| Nova Scotia | Prince Edward Island | 0.0009 | 0.399 | 0.345 |
| Nova Scotia | Quebec | 0.0009 | 0.468 | 0.513 |
| Nova Scotia | Saskatchewan | 0.0009 | 0.158 | 0.170 |
| Ontario | Prince Edward Island | 0.0009 | 0.302 | 0.302 |
| Ontario | Quebec | 0.0009 | 0.650 | 0.516 |
| Ontario | Saskatchewan | 0.0009 | 0.574 | 0.678 |
| Prince Edward Island | Quebec | 0.0009 | 0.430 | 0.501 |
| Prince Edward Island | Saskatchewan | 0.0009 | 0.117 | 0.157 |
| Quebec | Saskatchewan | 0.0009 | 0.658 | 0.694 |
| **PA level** |  |  |  |  |
| Active | Inactive | 0.0169 | 0.005 | 0.004 |
| Active | Moderate | 0.0169 | 0.134 | 0.059 |
| Inactive | Moderate | 0.0169 | 0.142 | 0.202 |

Northern Territories: Combined Yukon, Northwest and Nunavut Territories. PA level: Transportation and leisure physical activity level. NA=Not applicable.
